# Esmolol indirectly stimulates vagal nerve activity in endotoxemic pigs

**DOI:** 10.1186/s40635-018-0178-1

**Published:** 2018-07-04

**Authors:** Jerome Aboab, Louis Mayaud, Veronique Sebille, Rodrigo de Oliveira, Merce Jourdain, Djillali Annane

**Affiliations:** 1 Réanimation Polyvalente, Hôpital Raymond Poincaré, AP-HP, Université de Versailles Saint-Quentin-en-Yvelines (UVSQ), 104 bd. Raymond Poincaré, 92380 Garches, France; 2 Laboratoire d’ingénierie des systèmes de Versailles (LISV – UVSQ), 10-12 Avenue de l’Europe, 78140 Velizy, France; 3 EA 4275, Faculté de Pharmacie, Université de NANTES, 1, rue Gaston Veil, 44035 Nantes Cedex 1, France; 4 Laboratoire d’étude de la réponse neuroendocrine au sepsis, EA4342, Université de Versailles Saint-Quentin-en-Yvelines, 104, bd. Raymond Poincaré, 92380 Garches, France; 5 Service de Réanimation Polyvalente, Hôpital Roger Salengro, Rue Emile Laine, 59037 Lille, France; 6 Mensia technologies SA, 130 rue de Lourmel, 75015 Paris, France

**Keywords:** Animal experimentation, Endotoxemia, Septic shock, Autonomic nervous system, Adrenergic beta-antagonists, Cardiovascular diagnostic technic

## Abstract

**Background:**

There is an increasing interest in beta-blockade as a therapeutic approach to sepsis following consistent experimental findings of attenuation of inflammation and improved survival with beta1 selective antagonist. However, the mechanism of these beneficial effects remains very uncertain. Thus, this study is aimed at investigating the effects of a beta-1 selective blockade on sympathetic/parasympathetic activity in endotoxin-challenged pigs using heart rate variability. The hypothesis is that an adrenergic blockade could promote parasympathetic activity. Indeed, the increase of parasympathetic activity is a mechanism recently described as beneficial in septic states.

**Methods:**

Fifty-one endotoxin-challenged pigs were studied. After 30 min of endotoxin infusion and 30 min of evolution without intervention, the pigs were randomly assigned the placebo or esmolol treatment and were observed for 200 min. Overall heart rate variability was assessed continuously, in the temporal domain by standard deviation of RR intervals (SDNN, ms),and in the frequency domain by spectral powers of low frequency (LF, ms^2^ × 10^3^/Hz) and high frequency (HF, ms^2^ × 10^3^/Hz) bands.

**Results:**

Variations of power in these frequency bands were interpreted as putative markers of sympathetic (LF) and parasympathetic (HF) activity. In LPS treated animals, Esmolol did not increase SDNN, but instead decreased LF and increased HF power.

**Conclusion:**

These spectral modifications associated to a beta-blocker treatment after an endotoxemic challenge are interpreted as a significant decrease of sympathetic activity and an indirect increase of vagal autonomic tone.

**Electronic supplementary material:**

The online version of this article (10.1186/s40635-018-0178-1) contains supplementary material, which is available to authorized users.

## Background

In the recent years, beta-blockers—particularly beta-1 selective antagonists—have demonstrated promising effects in small [1] and large animals [2] with endotoxin/sepsis challenged [3–5]. In small animals, beta-blockade was associated with substantial reduction of pro-inflammatory mediators in tissue and blood [1, 3, 5], improvement in cardiac function [2, 3, 5], and increase in survival rate [3, 4]. The exact mechanisms underlying the favorable effects of a beta-blockade remain unclear. It could stem from the close interaction between the autonomic nervous system (ANS) and the immune system, which is now well recognized [6, 7].

A common way to accurately monitor ANS is the use of heart rate variability (HRV), which studies electrocardiographic (ECG) fluctuations. More precisely, the analysis of the time between two normal QRS complexes, i.e., RR intervals, reflects the parasympathetic and (ortho)sympathetic systems’ activities [8–11].

For instance, after the endotoxin challenge, impaired ANS function was found to be associated with reduction of the HRV in animals [12] as well as in healthy volunteers [13]. In septic patients, studies have also shown an association between pro-inflammatory mediators [14] and autonomic dysfunction captured by a reduced HRV [15, 16], which has been found to be associated with increased short-term mortality [16].

This link between autonomic function and inflammatory response paves the way to novel therapeutic approaches in the management of sepsis. For example, in animals challenged with endotoxin or with sepsis, stimulation of the parasympathetic system helped downregulate the inflammatory response to infection and therefore improved survival [7, 13, 17]. Concurrently, data has shown that using beta-blockers in porcine septic shock animal models was hemodynamically well tolerated [2] and could even improve mortality in patients [18].

Thus, the aim of this study was to investigate the effect of a selective β1 antagonist on HRV in endotoxin-challenged pigs. The hypothesis was that the antagonist allows for modulation and an eventual increase of parasympathetic activity.

## Methods

### Animals preparation

The study was approved by the Institutional Review Board for Animal Research and Care (University Department of Experimental Research-Lille-France and the ethical committee of experimental research of the CEA), and handling of the animals was in accordance with the National Institute of Health guidelines. Fifty-one piglets weighing 25 kg were anesthetized with an intramuscular injection of 2.5 mg/kg of body weight of ketamine (Ketalar; Parke-Davis, Courbevoie, France), followed by sodium pentobarbital (10 mg/kg of body weight).

The animals were intubated and mechanically ventilated (Evita 2 Dura, Luebeck, Germany). For all animals, the respiratory rate was set at 15 c/min. A catheter was inserted into the left carotid artery. The temperature was continuously monitored and kept at 38 °C by the use of heating lamps, suspended above the operating table.

### Hemodynamic parameters and recording of ECG for cardiovascular variability analysis

For HRV analysis, ECG and blood pressure (BP) waveforms were sampled at 500 Hz using an A/D converter (Biopac, System.inc, Paris, France) and continuously recorded and stored on a computer.

### Experimental protocol (Fig. 1)

After a stabilization period, animals were randomly assigned to the *Escherichia coli* lipopolysaccharide (LPS) or control group. LPS animals received a 30-min intravenous infusion of LPS (serotype 055:B5; Sigma Chemical Co., St. Louis, MO), diluted in 50 ml of sterile isotonic saline (LPS period from T0 to T+30 min). The concentration of LPS was 150 μg/kg. At T0+60 min, pigs were equally randomized to receive either a continuous intravenous infusion of esmolol (LPS-BB/CTRL-BB groups) or an equivalent volume of saline solution (LPS-CTRL / CTRL-CTRL) so that four experimental groups were formed. Esmolol was titrated to decrease heart rate by 10% compared with LPS animals in keeping with experiments in small animals [3, 5]. The introduction and adaptation procedure is detailed in the Additional file 1. Continuous sedation was achieved by propofol. Initially, a 15-mg propofol bolus was injected; after this induction, the flow rate was kept constant around 8 mg/kg/h, e.g., 200 mg/h. In practice, the flow was adapted according to the respiratory rate of the animal and possible movements. The doses used for each group are shown in Table 1. All animals were infused intravenously with isotonic saline throughout the entire study period in order to maintain a mean arterial pressure around 65 mmHg. The volumes infused for each group are shown in Table 1. None of the animals received vasopressors or inotropic drugs. Animals were euthanized with a bolus injection of pentothal (Dolethal Vetoquinol, Paris, France—200 mg/kg) at the end of the study.

**Fig. 1:**
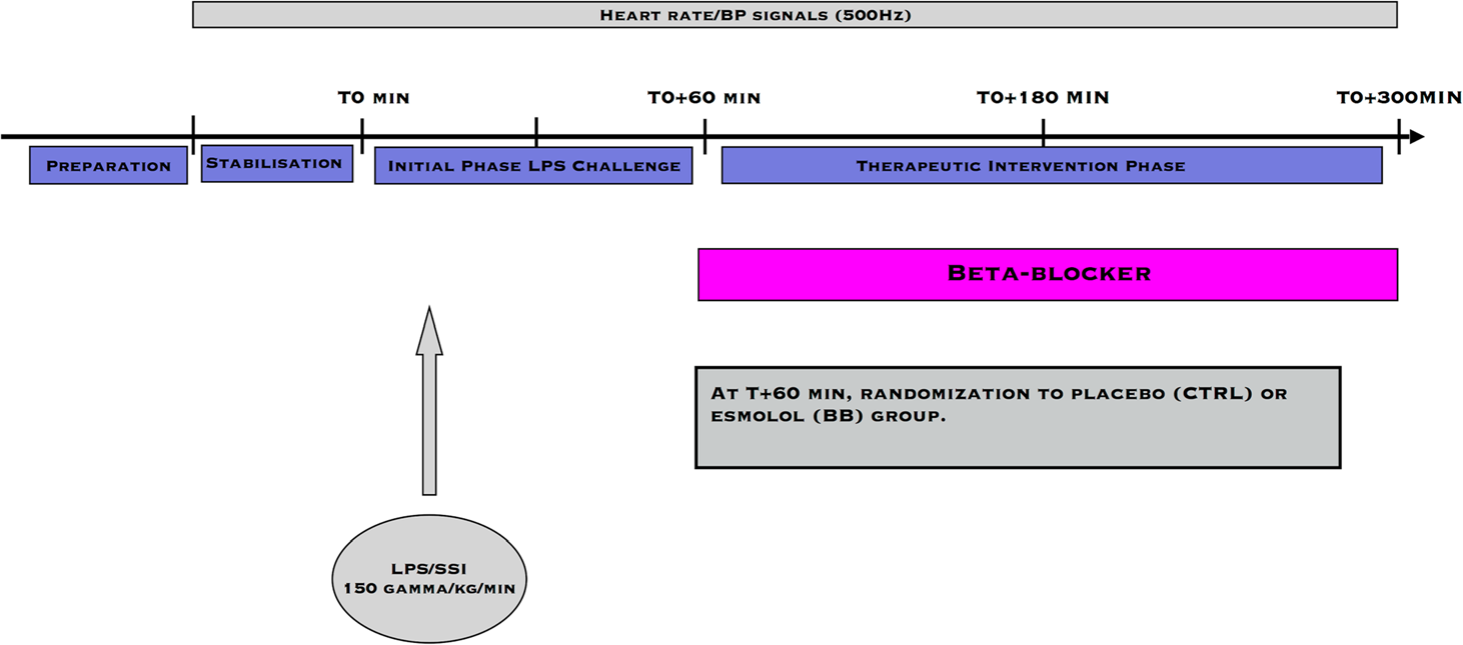
Study design. After a short period of stabilization, endotoxin was injected continuously during 30 min. Thirty minutes after the end of the LPS infusion beta-blocker was started

**Table 1:** Treatments during the experimentation

	CTRL-BB	CTRL-CTRL	LPS-BB	LPS-CTRL
Esmolol, microg/kg/min	166.7 ± 37	0	302.1 ± 30.6	0
Fluids, ml	772 ± 301	1044 ± 134	3514 ± 595	4028 ± 668
Propofol, mg	1105 ± 157	1232 ± 124	1212 ± 141	1225 ± 151

### Extraction of the RR intervals

For the HRV analysis, the data was processed using MATLAB (Mathworks, Natick, US). Each record was composed of BP and ECG sampled at 500 Hz for the length of the experiment and denoted *x*_abp_(*t*) and *x*_ecg_(*t*), respectively. The blood pressure time series was processed with wabp [19], a program developed to identify the systolic peak in each cardiac cycle from BP waveforms and made available open-source on the Physionet platform [20]. The detection of RR intervals was carried out using a custom R-peak detector inspired from Engelse et al. [21], where the raw ECG signal was derived, squared, and integrated. The resulting time series was used to detect peaks with an adaptive threshold obtained by sliding, forward and backward, a 5-s-long hamming window on the integrated signal. For each RR time series (derived from BP and ECG), outliers were identified in order to limit the influence of artifactual peak detection in subsequent analysis. Outliers were simply defined by any value that falls in one of the following groups: 1—RR intervals smaller than 10 ms; 2—greater than 5 s; 3—RR intervals departing from more than three standard deviations from the mean RR value estimated from a sliding windows of length 30 and 120 s, respectively; and 4- RR intervals for which the local first derivative defined as $$ {\displaystyle \begin{array}{l}i-1\\ {}t\\ {}\mathrm{RR}\left({t}_{\mathrm{i}+1}\right)-\mathrm{RR}\\ {}\frac{\mathrm{\partial RR}\left({t}_i\right)}{\partial t}=\end{array}} $$ is greater than three standard deviations from the mean estimated on the entire record. In total, for the ECG-derived RR intervals, 4.7% of all epochs were flagged as artefactual; the largest proportion of artefactual samples found in one file was 12.4%. The BP-derived RR interval time series were found to be of lesser quality with an overall proportion of artifacts of 17.4% and a maximum of 86.2% in one record. This work is therefore concentrated on the ECG-derived RR time series, while the BP-derived features were kept for consistency checks. Further study using this dataset could look at coupling between the ECG and the BP signal [22], which is why we made this dataset available online. Missing values were imputed with a third-order spline interpolation fired to each time series at the vicinity of imputed values so as to account for the temporal structure of the data.

### Heart rate variability analysis

**Fig. 2:**
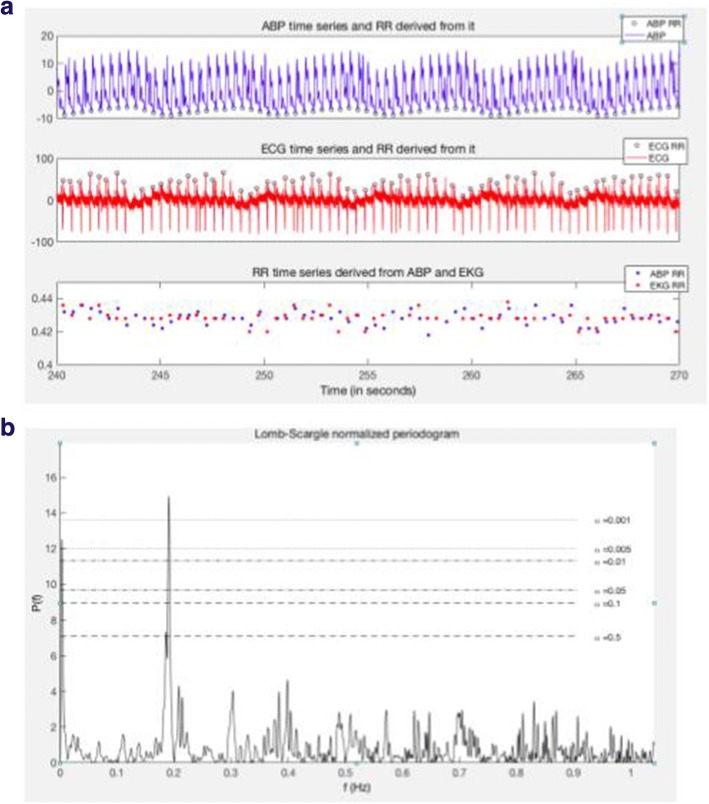
Heart rate variability. Temporal and spectral representation of 6-s-long ECG segment (*t* = 240 to 270 s) for pig 9 (file 25) showing: **a** the zero-centered ABP time series (blue in mmHg) with detected R peaks indicated with black circles, the zero-centered inverted ECG time series (red in mV) with detected R-peaks indicated with black circles and the resulting RR-interval time series for peak detection methods. **b** The Lomb-scale normalized perio-cardiogram derived from the ECG RR-interval time series showing the peak at 0.2 Hz induced by mechanical ventilation set at 15 cycles per minute

The RR interval time series were transformed to the spectral domain using the Lomb analysis and consecutive periods of 5 min of signal. The Lomb analysis was preferred over the more traditional Fast Fourier Transform (FFT) because it naturally handles the non-evenly sampled nature of the RR intervals thereby discarding the need of a potentially deteriorating resampling step. This spectral analysis was then used to quantify energies within the very low frequency (VLF), low frequency (LF), and high frequency (HF) bands defined between 0.0033, 0.04, 0.15, and 0.4 Hz [23], respectively. These were ultimately combined to extract several features over each 5-min segment of the data: VLF, LF, HF, their normalized version (divided by VLF+LF+HF), and the ratio LF to HF. The mean RR and its standard deviation (STD) were also computed from the same time window. The extraction of these features was carried out on non-overlapping 5-min-long RR interval time series [24]. Typical plots for the temporal and spectral analysis are presented on Fig. 2. As a consequence, each recording consisting of one ECG waveform sampled at 500 Hz was transformed onto eight features sampled every 5 min.

### Statistical analysis

An H-max randomization method was implemented to identify statistical significance. For this analysis, groups were permuted under the null hypothesis so that no difference in time series was to be observed between groups; an arbitrary chosen number of permutations *k* = 1e4 was taken at random from all the $p=\frac{\mathrm{n}!}{\mathrm{k}!}$ possible permutations. For each permutation *p*_*i*_ and for each variable *v*_*j*_, a statistical time series ${H}_i^j(t)$ was obtained by computing a Kruskall-Wallis test comparing the value distributions at every time *t*. An H-max distribution was computed by taking the maximum values of ${H}_i^j(t)$ across all variables and time: $\left\langle {H}_i\right\rangle {=\max}_{j,t}{H}_i^j(t)$. The 95th percentile of this distribution, *τ*_95th_, was used as a threshold for statistical significance and compared to the actual values of observed statistics ${H}_i^j(t)$. The Hmax distribution was then iteratively refined by removing all ${H}_i^j\left({t}_k\right)$ values where *t*_*k*_ denotes the time when ${H}_{\mathrm{i}=0}^j\left({t}_k\right)>{\uptau}_{95\mathrm{th}}$, meaning that at least one variable *v*_*j*_is found significant for the non-permuted time series. This leads to a new estimate of the threshold of significance ${\tau}_{95\mathrm{th}}^m$. The process was repeated until the relative change in the threshold ${\Delta \uptau}_{95\mathrm{th}}^m=\frac{\tau_{95\mathrm{th}}^m}{\tau_{95\mathrm{th}}^{m-1}}$ was found smaller than 5%.

## Results

**Fig. 3:**
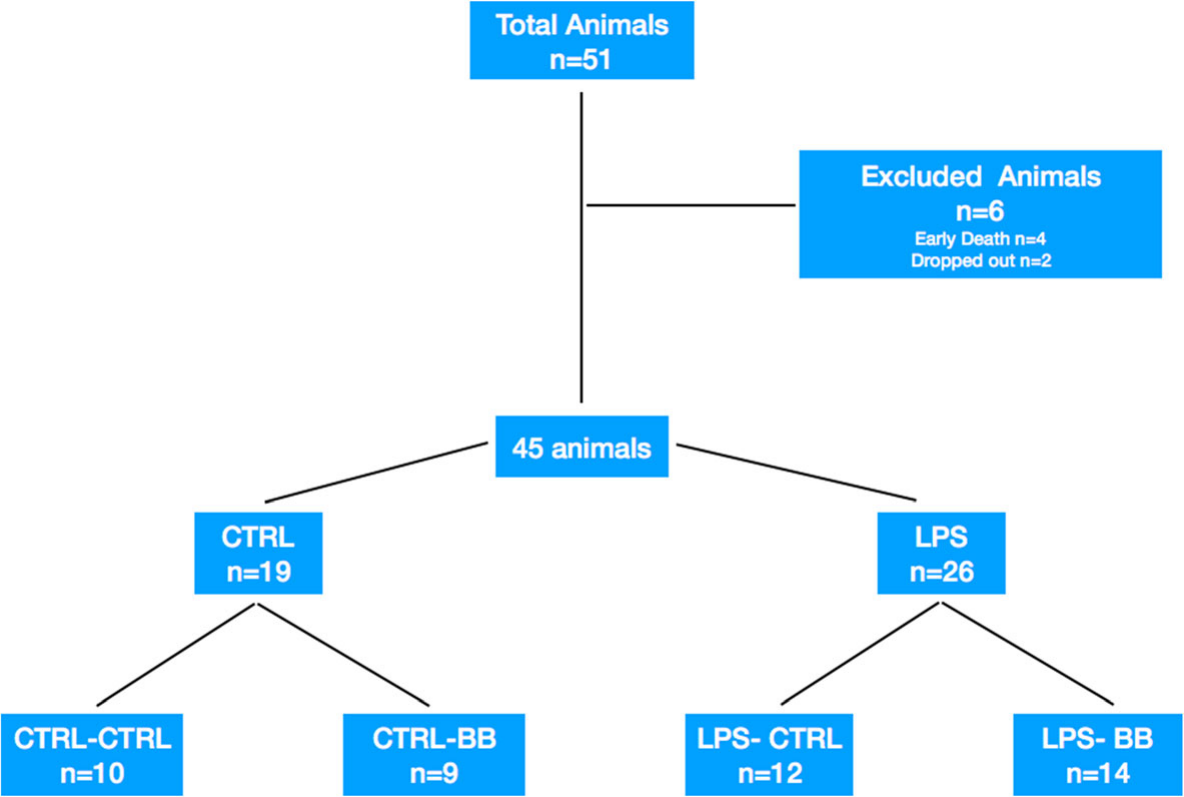
Flow chart. Fifty-one animals were included in the study and six were excluded before randomization

A total of 51 animals were included in the study. Six animals died without the experimentation being completed: two late deaths in the LPS group and four early deaths due to difficult conditioning (Fig. 3).

### Comparison of CTRL-* (*n* = 19) and LPS-* (*n* = 26) groups during initial phase of LPS challenge (T0+30 to T0+90 min)

**Fig. 4:**
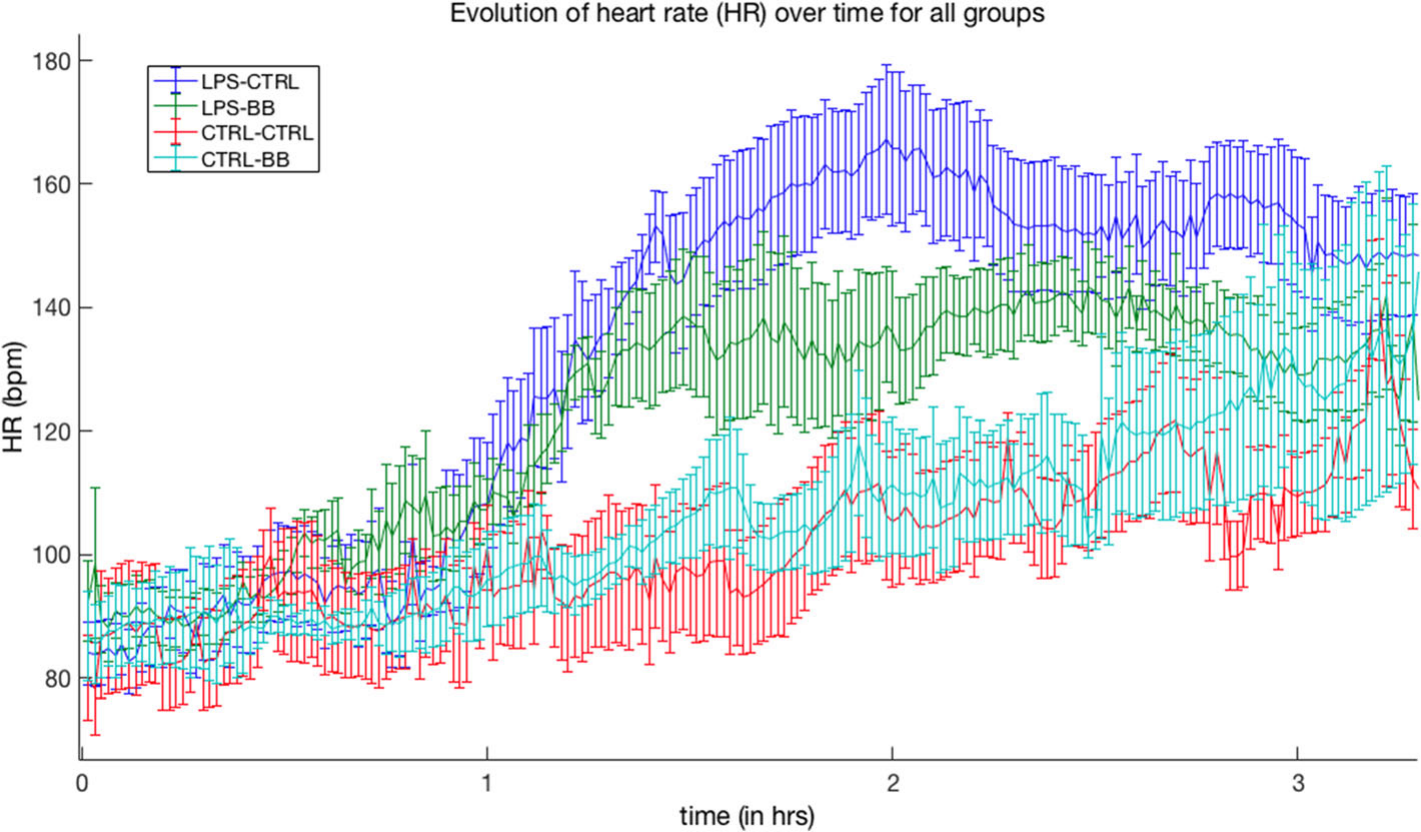
Evolution of heart rate. Evolution of heart rate over the course of the experiment (*t* = 0 to 3 h) for all experimental groups: LPS-CTRL (blue), LPS-BB (green), CTRL-CTRL (red), and CTRL-BB (cyan). Each point represents a 5-minute sample epoch from which the median and interquartile ranges are derived and indicated with error bars. Starting from 1 hour, the LPS-* groups start showing increased HR following endotoxin challenge. Shortly before 2 h, the LPS-BB shows a reduced HR compared to the LPS-CTRL group mediated by beta-blocking effect

After the LPS infusion, heart rate increased by + 44% on average and 11% in the CTRL-* group (LPS-* period) (*p* < 0.05 LPS-* vs CTRL-* from file 10 to 90 min) (Fig. 4). During this first phase, no modification in variability analysis appears (detailed data are available in Additional file 1*:* Figure S1).

### Comparison of LPS-CTRL (*n* = 12) and LPS-BB (*n* = 14) during therapeutic intervention (T0+90 min to the end)

**Fig. 5:**
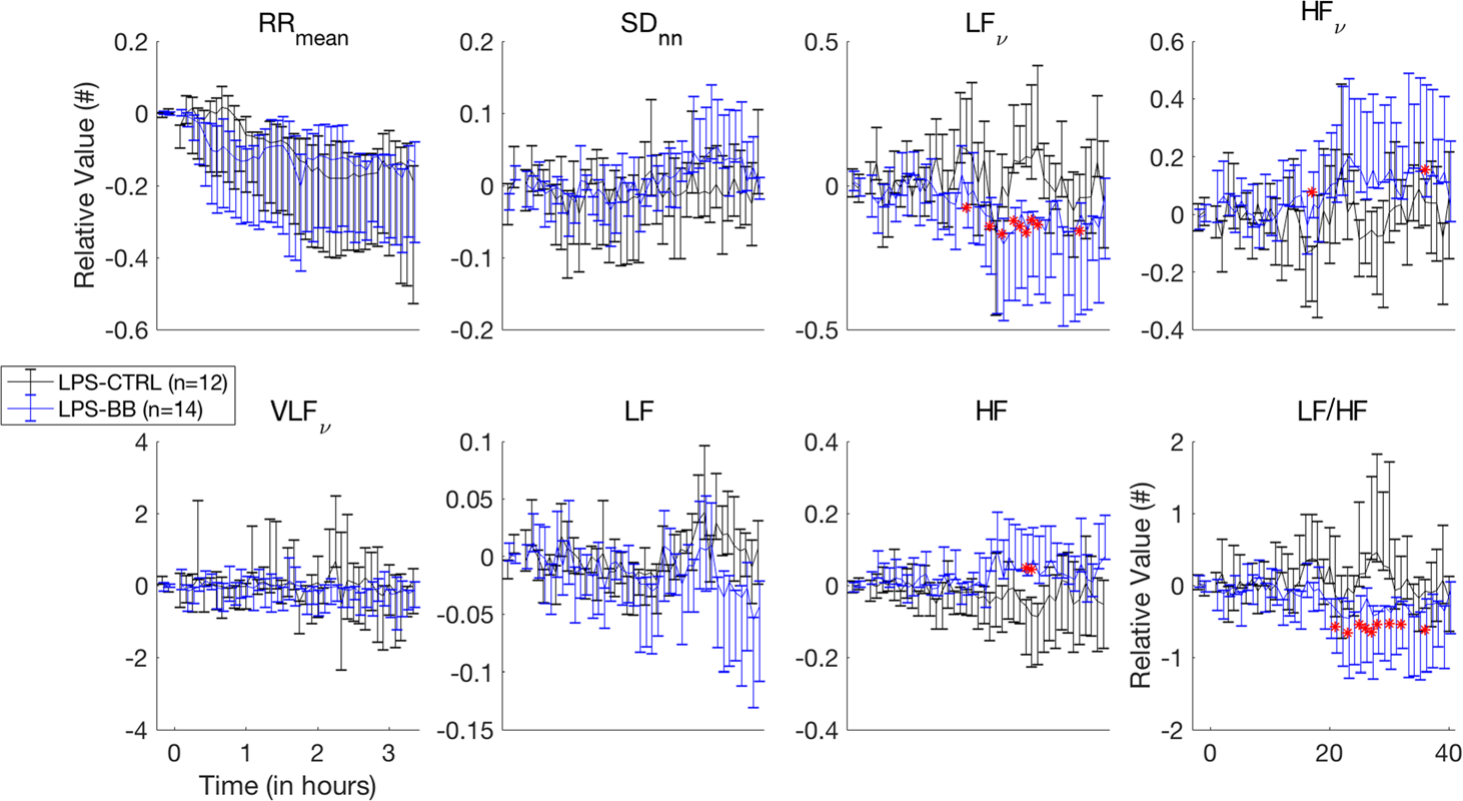
Temporal and spectral evolution in LPS-BB/LPS-CTRL groups. Evolution of temporal and spectral parameters over the course of the experiment (*t* = 0 to 3 h) for LPS-BB (blue) and LPS-CTRL (black) groups: each point represent a 5-min sample from which the median and interquartile ranges are derived and indicated with error bars. There appears to be a trend towards increasing SD in the LPS-BB group. Spectral analyses show a drop in spectral power for the LF band as it increases for the HF band. This is characterized by a clear decrease in values for the HFLF ratio for the LPS-BB group compared to the LPS-CTRL group

During this period, the dosage of esmolol was adapted to obtain a decrease in HR of 10% (Fig. 4). The mean BP was not different between the two groups. Also, there appears to be a slight increase of SDNN in the LPS-BB group compared to the LPS-CTRL group. This was associated with a very significant drop in the LF band and an increase in the HF (Fig. 5).

### Comparison of CTRL-CTRL (*n* = 10) and LPS-CTRL (*n* = 12) groups during therapeutic intervention (T0+90 min to the end)

**Fig. 6:**
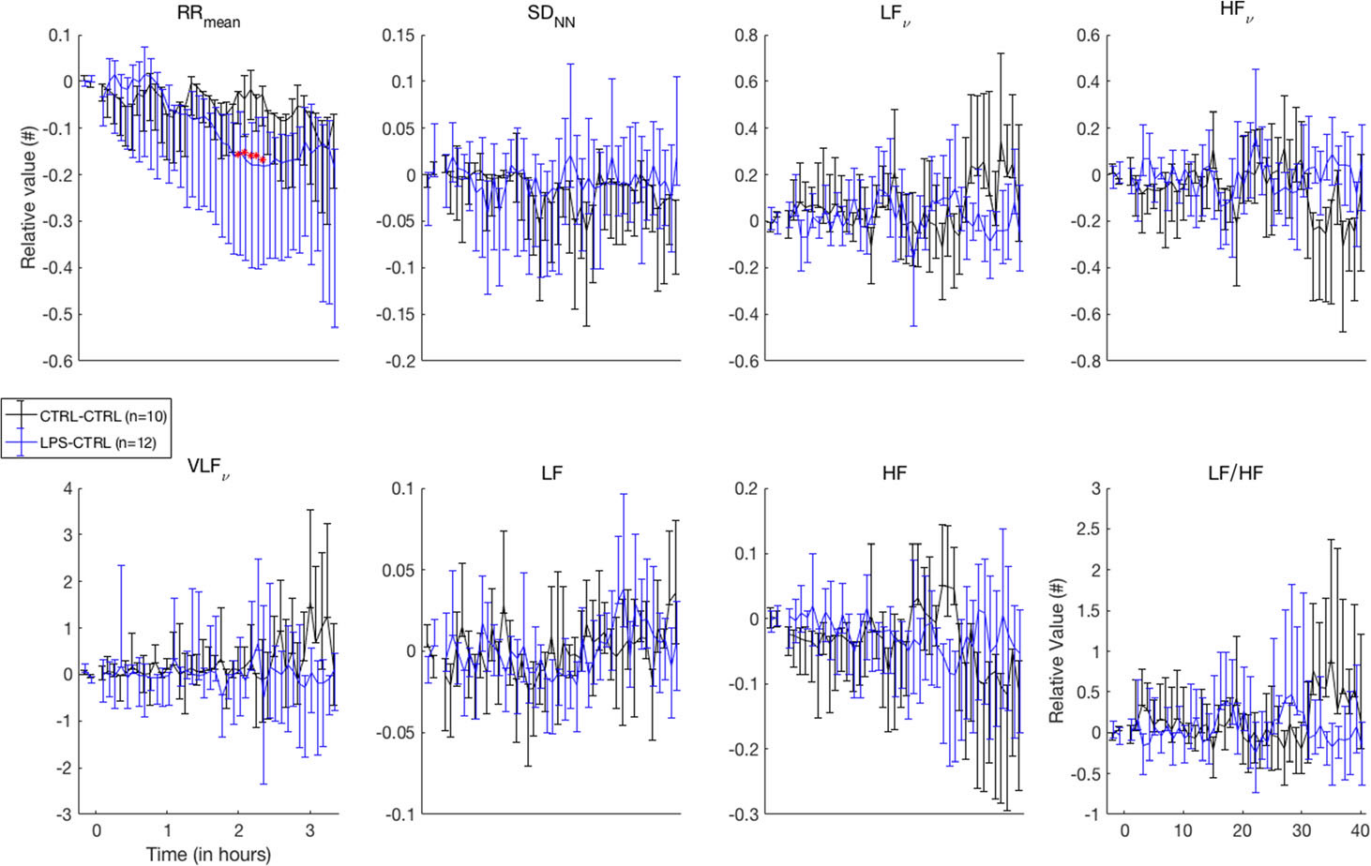
Temporal and spectral evolution in LPS-CTRL/CTRL-CTRL groups. LPS injection was not associated with any change in temporal and spectral indexes

After the initial phase, the HR remained stable in the LPS-CTRL group and increases only by 20% from the 90 min to the end of the experiment and increased regularly in the CTRL-CTRL group. Simultaneously, the ABPm decreased drastically and stabilized at a much lower level than in the CTRL-CTRL group. No difference of variability was observed (Fig. 6).

### Comparison of CTRL-CTRL (*n* = 10) and CTRL-BB (*n* = 9) groups during therapeutic intervention (T0+90 min to the end)

The esmolol dosage used for the HR drop in LPS animals failed to obtain a frequency drop in the CTRL-BB group compared with the CTRL-CTRL group. The mean BP remained stable in both groups during the experiment. At these hemodynamic conditions, esmolol administration was associated with an increase of SDNN without other effects on spectral parameters (detailed data are available in Additional file 1*:* Figure S2).

### Comparison of CTRL-BB (*n* = 9) and LPS-BB (*n* = 14) groups during therapeutic intervention (T0+90 min to the end)

The HR is much higher in the LPS-BB group compared to the CTRL-BB group. The BP is comparable. SDNN clearly decreased in the LPS-BB compared to the CTRL-BB group. In terms of spectral parameters, LF decreased and HF increased in the LPS-BB group in comparison with CTRL-BB (detailed data are available in Additional file 1*:* Figure S3).

## Discussion

The primary difficulty of this work was to experimentally and technically isolate the variations of the ANS revealed by the HRV analysis, namely, the preparatory surgical procedure, the endotoxin challenge, the experimental conditions (filling, ventilation, heat), and the therapeutic intervention (beta-blockers). Indeed, the ANS is an integrative system, and its activity can be modified by countless stimuli such as pain, cold, and light so that HRV interpretation is a greatly challenging task that requires a strict experimental control. To that effect, the animals’ core temperature was kept constant by heating lamps; animals were ventilated at a fixed respiratory rate (15 cycles per minute) and tidal volume.

Another limitation of this work lies in the animal model that can only partly extrapolate to the human physiology. In order to limit this bias, we used a pig model for its proximity to humans in cardiac function and parameters. This porcine model was previously shown to result in reproducible cardiovascular alterations after the endotoxin challenge [2].

One of the results of this study is the absence of a typical HRV response to the endotoxin challenge. Indeed, the endotoxin aggression did not significantly alter the measured spectral components (Additional file 1: Figure S1). This observation supports the difficulty to demonstrate a specific pattern of spectral data associated with septic aggression, which somehow contradicts previous reports [13–16]. A possible explanation for this discrepancy with literature is the use of an anesthetic drug. In our study, the animals were sedated with propofol, which is known to reduce the activity of the ANS [25], even though its effect is believed to be smaller than that of other currently used sedative drugs [25]. In studies that report changes in HRV during sepsis or other conditions, it is noted that sedation conditions are rarely specified [14–16]. Arguably, a more precise look at the conditions of sedation and its normalization across subjects is recommended for future studies investigating HRV. Usually, the non-septic population beta-blockers are supposed to increase the SDNN [26], which was observed in the CRTL-* but not in the LPS-* groups. In other words, the effect of beta-blockers on SDNN could only be observed in the non-challenged animals. The high heart rate could explain this result [27] by a mechanical effect. Indeed, in the CTRL-BB population, the heart rate was lower compared to the LPS groups, and the SDNN was significantly increased compared to CTRL. This illustrates the possible mechanical effect correlating the high HR with a low SDNN [27].

To our knowledge, this study is one that has included some of the largest study population, which allows us to obtain a satisfactory statistical power. It also benefits from a strong experimental protocol in which we tried to control for many confounding factors possibly affecting ANS activity: temperature, filling, mechanical ventilation, sedation, and intervention. Hemodynamic analyzes are longitudinal throughout the entire experiment, giving a complete picture on the evolution of the cardiovascular system. Furthermore, we used cutting edge techniques to carry out the HRV analysis [22] with a dedicated care to signal quality and artifact rejection. The analysis was performed using the open-source toolbox, which increases replicability of our work. To that effect, we have also made our dataset freely available.

In addition to this, the SDNN power is relatively low in all the animal groups as reported in other studies [28]. This gives a strong importance to the spectral variations observed during the endotoxemic aggression. Indeed, despite the lack of difference between LPS and LPS-BB groups in the time domain, there was a significant difference observed in the frequency domain (Fig. 3). The other element is the interrelationship and balance between the spectral power in the LF and HF bands when the level drops in one it increases in the other and vice versa.

During the endotoxemic challenge, the adrenergic blocking did not change the global variability, approached by the SDNN. This is in contradiction with other reports where septic challenge is usually associated with SD decrease [12, 15]. In this experiment, the aggressiveness of the procedure (catheter placement, intubation) as well as sedation most likely explains this result.

Conversely, a spectral power reduction in the LF band and an increase in the HF band were observed (Fig. 3) in the LPS-BB as compared to the LPS-CTRL group during initial endotoxemic periods. In CTRL-CTRL animals, esmolol seemed to slightly increase the SDNN power, but no difference was observed for the spectral parameters (Additional file 1: Figure S2).

What are the potential implications of these findings? Cardiovascular variability in essence is a balance between the sympathetic and the parasympathetic modulation of the system. In our study, the blockade of cardiac beta sympathetic activity by a beta-blocker results in an increase in the LF/HF ratio, which actually reflects a relative increase in para-sympathetic activity. This esmolol-induced effect on the ANS may actually explain the benefits from beta1 selective antagonists observed on cardiac function [5] and on survival [4] in endotoxin-challenged or in septic animals.

Indeed, the observed increase in parasympathetic activity is consistent with increase in vagus nerve activity. Owing to the well-established anti-inflammatory activity of the parasympathetic system [7], these findings suggest that previously demonstrated anti-inflammatory effects of esmolol in endotoxin-challenged [1] and septic animals [3–5] are at least partly related to enhanced vagal activity.

## Conclusion

Infusion of esmolol may be the first clinically relevant mean to indirectly stimulate the vagus nerve in patients with sepsis. This observation could be one explanatory element of the positive effect observed in septic shock human [18].

## Additional file

Additional file 1: Supplemental digital content. (DOCX 310 kb)

## Abbreviations

ANS: Autonomic nervous system
BP: Blood pressure
ECG: Electrocardiographic
FFT: Fast Fourier Transform
HF: High frequency
HRV: Heart rate variability
LF: Low frequency
LPS: Lipopolysaccharide
SDNN: Standard deviation of RR intervals
VLF: Very low frequency
