# Correction to: Esmolol indirectly stimulates vagal nerve activity in endotoxemic pigs

**DOI:** 10.1186/s40635-018-0191-4

**Published:** 2018-08-20

**Authors:** Jerome Aboab, Louis Mayaud, Veronique Sebille, Rodrigo de Oliveira, Merce Jourdain, Djillali Annane

**Affiliations:** 1 Réanimation Polyvalente, Hôpital Raymond Poincaré, AP-HP, Université de Versailles Saint-Quentin-en-Yvelines (UVSQ), 104 bd. Raymond Poincaré, 92380 Garches, France; 2 Laboratoire d’ingénierie des systèmes de Versailles (LISV – UVSQ), 10-12 Avenue de l’Europe, 78140 Velizy, France; 3 EA 4275, Faculté de Pharmacie, Université de NANTES, 1, rue Gaston Veil, 44035 Nantes Cedex 1, France; 4 Laboratoire d’étude de la réponse neuroendocrine au sepsis, EA4342, Université de Versailles Saint-Quentin-en-Yvelines, 104, bd. Raymond Poincaré, 92380 Garches, France; 5 Service de Réanimation Polyvalente, Hôpital Roger Salengro, Rue Emile Laine, 59037 Lille, France; 6 Mensia technologies SA, Paris, France

## Correction

Following publication of the original article [1], the author reported these required corrections to Fig. 5 and Fig. 6:

Fig. 5:

The last graph of the first line, titled LF v, should be **HFv**

The last bottom graphs, titled SDnn and RR nn, should be **HF** (instead of SDnn) and **LF/HF** (instead of RRnn)

Fig. 6:

The title of the last graph bottom line is **LF/HF** (instead of HF/LF).

The original article [1] has been updated to reflect this.
