# Supplementary material for: Esmolol indirectly stimulates vagal nerve activity in endotoxemic pigs
Source: Intensive Care Med Exp. 2018 Jul 4;6:14. doi: 10.1186/s40635-018-0178-1 (PMC6031554; doi:10.1186/s40635-018-0178-1)

**SUPPLEMENTAL DIGITAL CONTENT**

**Esmolol Indirectly Stimulated Vagal Nerve Activity in Endotoxemic Pigs**

**Jerome ABOAB** ***^$^; Louis MAYAUD ^$^; Veronique** **SEBILLE** *****; Rodrigo** **de OLIVEIRA** ****; Merce JOURDAIN ^$ $^; Djillali ANNANE** *^$^

**Affiliation:**

* Réanimation Polyvalente, Hôpital Raymond Poincaré, AP-HP, Université de

Versailles Saint-Quentin-en-Yvelines (UVSQ), 104 bd. Raymond Poincaré, 92380 Garches, France

** Laboratoire d’ingénierie des systèmes de Versailles (LISV – UVSQ); 10-12 Avenue de l’Europe, 78140 Velizy, France

*** EA 4275, Faculté de Pharmacie, Université de NANTES, 1, rue Gaston Veil, 44035 Nantes Cedex 1, France

**^$^** Laboratoire d’étude de la réponse neuroendocrine au sepsis, EA4342, Université de Versailles Saint-Quentin-en-Yvelines, 104, bd. Raymond Poincaré, 92380 Garches, France

**^$$^** Service de Réanimation Polyvalente, Hôpital Roger Salengro, rue Emile Laine, 59037 Lille, France

**^$$$^** Mensia technologies SA, 130 rue de Lourmel, 75015 Paris

**Corresponding Author:**

Dr Jérôme Aboab , Service de Réanimation Polyvalente

Hôpital Raymond Poincaré (AP-HP) 104, bd. Raymond Poincaré, 92380 Garches, France

phone 1: +33 6 11 94 88 90

phone 2: +1 617 852 1895

E-mail: jerome.aboab@gmail.com

**Procedure to introduce Esmolol**

The recommandation to use esmolol includ 4 levels ranging from 50 to 200 μg / kg / min. Each level consists of a loading dose of 500 μg / kg / min to infuse in 1 minute followed by a maintenance dose of 50, 100, 150 and 200 μg / kg / min. Efficacy is assessed after the first 4 minutes of infusion. If the goal is reached the infusion rate is continued. If the effect on the heart rate is insufficient, it is necessary to go to the next level. In order to minimize the risks the procedure of introduction of the esmolol was modified as follows: (1) no bolus was realized, (2)first step at 100 μg / kg / min, (3) duration of each step of 10 min, (3) the increment between each level was fixed at 100 μg / kg / min.

**Procedure for adjusting esmolol**

If a blood pressure decrease of more than 15% was observed , the dosage of esmolol was reduced in increments of 50 μg / kg / min every 5 minutes until restoration of initial blood pressure.

**os Fig1**


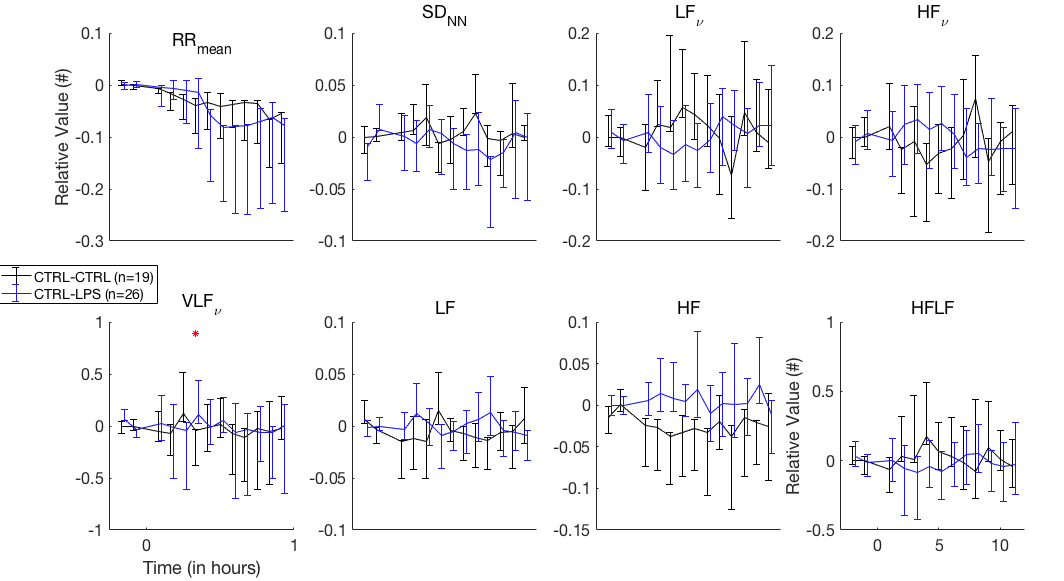


**os Fig 2**


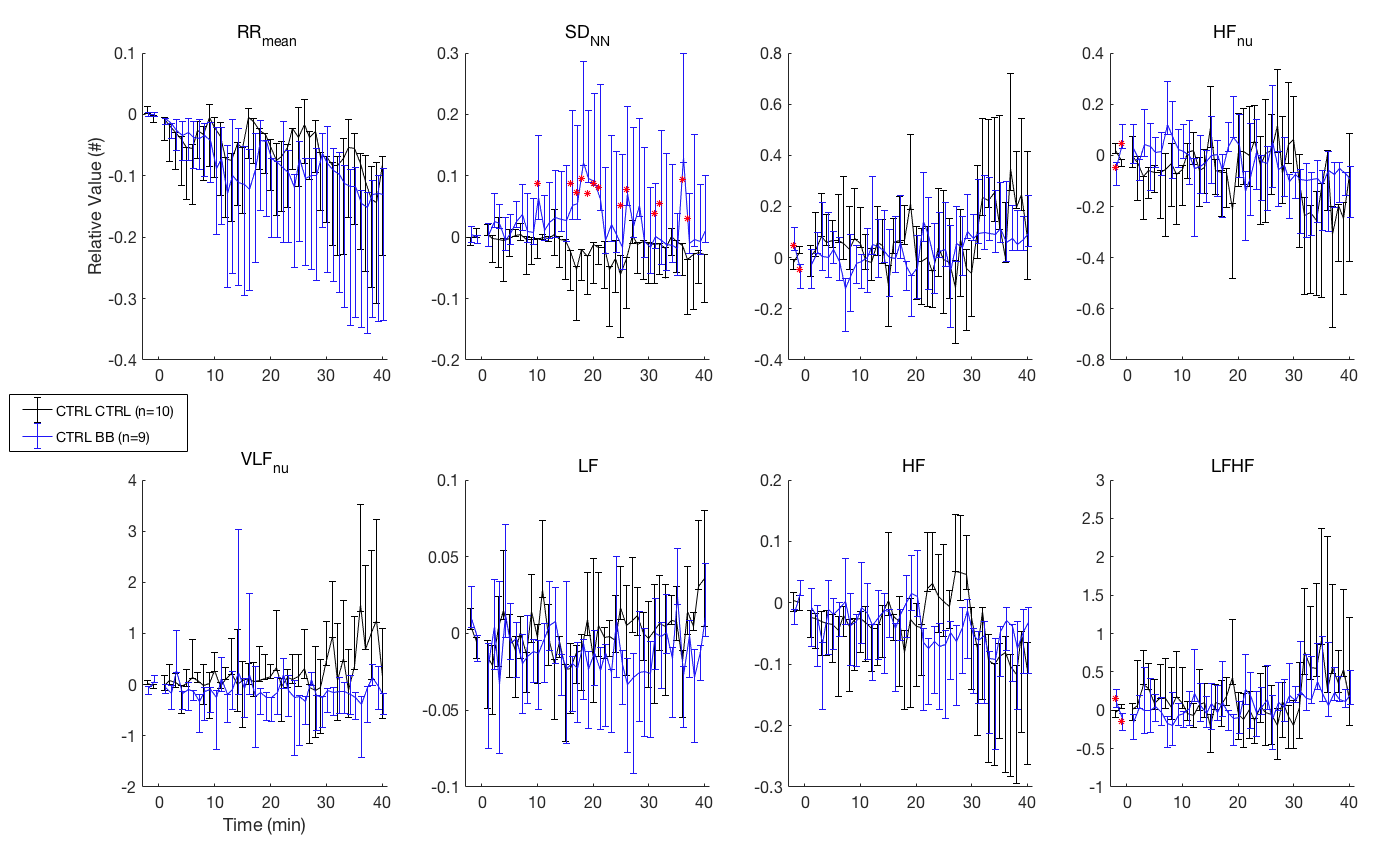


**osFig3**


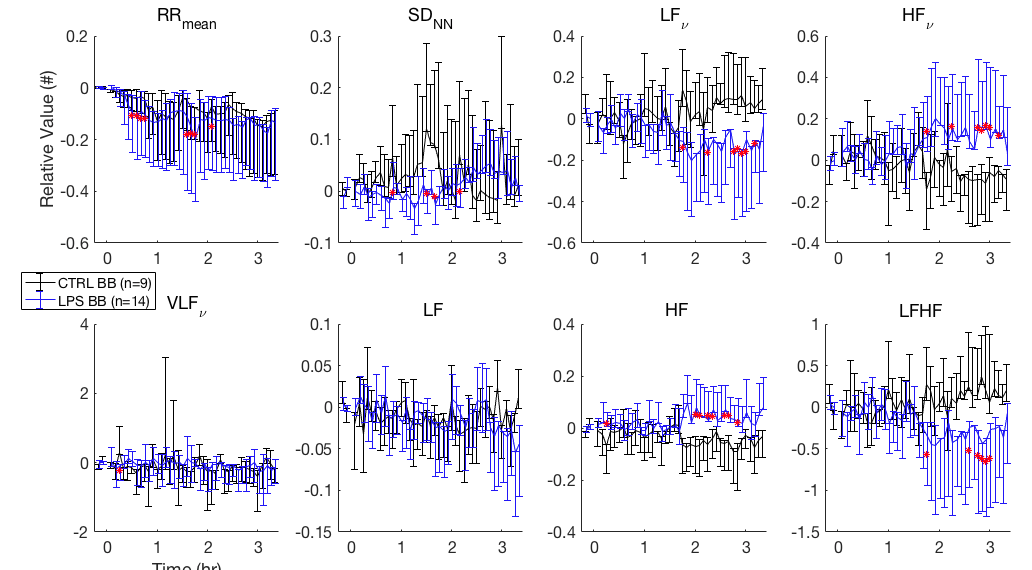

Supplement: Supplementary file 1 — Supplemental digital content. (DOCX 310 kb) [file 40635_2018_178_MOESM1_ESM.docx]
